# Study of Nonlinear Models of Oscillatory Systems by Applying an Intelligent Computational Technique

**DOI:** 10.3390/e23121685

**Published:** 2021-12-15

**Authors:** Naveed Ahmad Khan, Fahad Sameer Alshammari, Carlos Andrés Tavera Romero, Muhammad Sulaiman

**Affiliations:** 1 Department of Mathematics, Abdul Wali Khan University Mardan, Khyber-Pakhtunkhwa 23200, Pakistan; ahmednaveed854477@gmail.com; 2 Department of Mathematics, College of Science and Humanities in Alkharj, Prince Sattam bin Abdulaziz University, Al-Kharj 11942, Saudi Arabia; 3 COMBA R&D Laboratory, Faculty of Engineering, Universidad Santiago de Cali, Cali 76001, Colombia; carlos.tavera00@usc.edu.co

**Keywords:** nonlinear oscillator, mass attached to a stretched elastic wire, large amplitude, damping, Runge–Kutta method, neural networks, Levenberg–Marquardt algorithm, soft computing

## Abstract

In this paper, we have analyzed the mathematical model of various nonlinear oscillators arising in different fields of engineering. Further, approximate solutions for different variations in oscillators are studied by using feedforward neural networks (NNs) based on the backpropagated Levenberg–Marquardt algorithm (BLMA). A data set for different problem scenarios for the supervised learning of BLMA has been generated by the Runge–Kutta method of order 4 (RK-4) with the “NDSolve” package in Mathematica. The worth of the approximate solution by NN-BLMA is attained by employing the processing of testing, training, and validation of the reference data set. For each model, convergence analysis, error histograms, regression analysis, and curve fitting are considered to study the robustness and accuracy of the design scheme.

## 1. Introduction

The study of nonlinear problems is of great importance in all areas of applied mathematics, engineering, and physics. Most of the phenomena occurring in these fields are modeled as nonlinear differential equations. In general, exact or analytical solutions of highly nonlinear differential equations do not always exist and hence most of the researchers have used either approximate analytical techniques or numerical methods to obtain approximate solutions. Only a few nonlinear systems can be directly solved; thus, numerical methods, particularly the well-known Runge–Kutta method of the fourth order, are commonly employed to derive approximate solutions [1]. Nonlinear oscillators are considered fundamental equations that have gained the attention of researchers, and many methods have been used to find approximate and numerical solutions to various nonlinear oscillators [2,3,4]. Ji-Huan [5] proposed a new perturbation in contrast to other traditional perturbation techniques for the solution of the Duffing equation with a high order of nonlinearity. In their study, they constructed the homotopy with an imbedding parameter *p*, which is used as a small parameter. Oliveira in [6] deal with the problems in nonlinear mechanics by using the method of averaging. They discuss the developments of the method of averaging and construct the approximate solutions for oscillatory models with small and large parameters. Amol Marathe [7] investigated the attenuation of harmonic waves through a periodic and discrete structure with a frequency ostensibly within the propagation zone due to mild damping. They used the matrix transform method and adopted harmonic balance to study the linear and nonlinear damping. Hui Li [8] developed an energy balance approach for calculating the frequency–amplitude relationships of nonlinear oscillators with large nonlinearities and discontinuous terms. M. Heydari [9] combined the spectral method and variational iteration method to investigate the famous strongly nonlinear oscillators. Liu Ming studied the oscillations in a pendulum by modifying the Adomian decomposition method (ADM) [10].

In recent years, different nonlinear oscillatory problems have gained the attention of the research community due to the stiffness and rigidness in the behavior of the mathematical models [11,12]. Akuro [13] investigated the periodic oscillation and performed bifurcation analysis of a pendulum with spinning support using the modified continuous piecewise linearization method. Kargar [14] used He’s Energy Balance Method (HEBM) and He’s Amplitude-Frequency Formulation method (HFAF) to perform the frequency analysis of a rotational pendulum system with large-amplitude oscillation. Nonlinear oscillation typified as a mass attached to an elastic wire was studied by [15]. S. Li [16] used the harmonic balance method to analyze the Duffing oscillation of van der Pol oscillators. An analysis of large-amplitude oscillations in triple wells of a non-natural system was conducted by S. Lai [17]. Razzak [18] studied the phenomena of Duffing oscillators with rational and irrational forces, while A. Koochi [19] investigated the nonlinear oscillations of a CNT Nano-resonator based on nonlocal electricity using the energy balance method. Qian and Liu [20,21] used the residue harmonic method to study the vibrations of a system of cantilever beams carrying an intermediate lumped mass. All the above-discussed techniques were developed to analyze the mathematical models under different scenarios.

Oscillators are extremely important in physics because of their mathematical properties as expressed in the Fourier theorem. It represents the regular periodic change in a system. Oscillations occur not only in mechanical systems but also in dynamical systems and virtually in every area of science—for example, the periodic firing of nerve cells in the brain, the beating of the human heart (for circulation), predator–prey population cycles in ecology, business cycles in economics, the vibration of strings in guitars and other string instruments, geothermal geysers in geology, and the periodic swelling of Cepheid variable stars in astronomy. Testing and correctly interpreting the results of such nonlinear oscillatory systems is inherently complex. Preconceived notions about how the system would respond must be avoided at all costs, as this will often influence the types of tests and processing techniques used. Due to the nonlinear and stiff nature of the mathematical model of nonlinear oscillators, a number of semi-analytic methods, such as the iteration perturbation method (IPM), modified differential transform method (MDTM) [22], max-min approach [23], linearized perturbation method (LPM) [24], modified simple equation (MSE) scheme [25], Lindstedt–Poincare method [26], extended BKP–Boussinesq equation [27], and homotopy analysis method (HAM) [28], have been developed. Besides their advantages, these techniques are deterministic approaches and mostly require prior information about the gradient and other essential parameters. In this study, a stochastic approach based on supervised machine learning is utilized to find numerical solutions for various nonlinear oscillators. These stochastic algorithms have various applications in different areas, including biomathematics [29], civil engineering [30], petroleum engineering [31], wireless communications [32], electrical engineering [33,34], and wire coating dynamics [35]. These facts and their significance inspired the authors to exploit and explore the architecture of neural networks to solve stiff and strongly nonlinear models of oscillators. Some potential outcomes of the given study are summarized below.

- The main purpose of this study is to formulate mathematical models and investigate the influence of variations in certain parameters of nonlinear oscillators such as a rotational pendulum system, mass attached to an elastic wire, a uniform beam carrying an intermediate lumped mass, a two-mass system with three springs, the van der Pol equation, and a two-mass system with small damping.
- An integrated novel design of soft computing based on neural networks and the backpropagated Levenberg–Marquardt algorithm is utilized to study the displacement, velocity, and acceleration of the models.
- The supervised learning of the NNs-BLM algorithm works effectively on the data set generated by a numerical solution using the Runge–Kutta method.
- The performance of the design scheme is validated by conducting convergence analysis based on mean square error, regression analysis, error histogram, and curve fitting with reference data. Results demonstrate that the proposed algorithm is smooth and easy to implement.

## 2. Proposed Methodology

**Figure 1:**
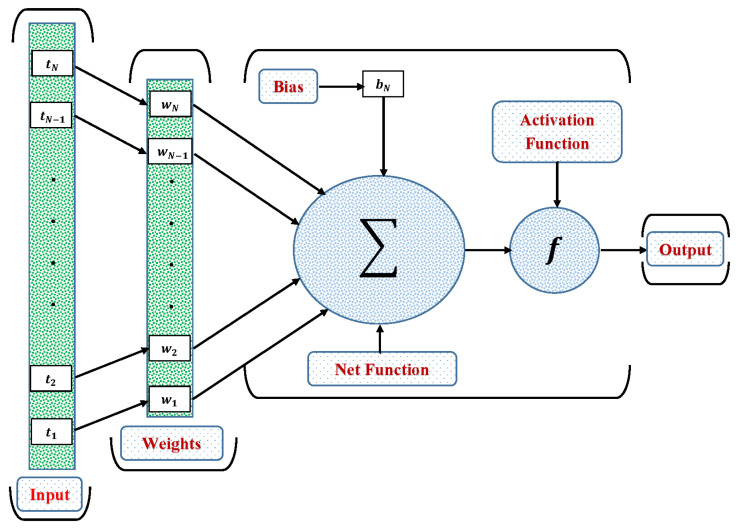
Architecture of the basic ANN.

In this section, a novel machine learning technique based on the supervised learning of neurons in artificial neural networks (ANNs) is utilized to study the oscillations in various systems. The control of oscillations in nonlinear systems is a serious challenge for engineers. The destabilization in the oscillatory systems can create a serious thread. Therefore, an automatic oscillation detection tool is required to quickly detect the frequency and amplitude of oscillations in the systems. In this work, a new approach based on machine learning for periodic solutions of the oscillatory systems has been proposed using ANNs, in a multi-layer perceptron (MLP) configuration. The MLP, also known as the Feed-Forward Neural Network (FNN), is a type of neural network that has a hidden layer between the input and output layers. The ANN controller for a single neuron is shown in Figure 1. Mathematically, an ANN model can be written as

(1) $$Y=f\left[v_{o}+\sum_{j=1}^{m}h\left(\lambda_{j}+\sum_{i=1}^{n}x_{i}w_{ij}\right)v_{j}\right]$$

where *Y* and $x_{i}$ are the output and input data, $v_{o}$ is the output bias, *m* and *n* denote the number of hidden and input neurons, respectively, $\lambda_{j}$ is the hidden unit biases, and $w_{ij}$ are weights. *h* and *f* are activation functions at the hidden input layer and hidden output layer, respectively. In this study, Log-sigmoid is used as an activation function between the hidden and output layers, which is given as

(2) $$f\left(x\right)=\frac{1}{1+e^{-x}},$$

Further, the Feed-Forward Neural Network is optimized with a training algorithm such as the backpropagated Levenberg–Marquardt algorithm. It is also known as the damped least-squares (DLS) method and is used to minimize the nonlinear minimization problem by using the least square fitting. The LM algorithm is used as a built-in function in various applications to find a local minimum. It interpolates between the method of gradient descent (GD) and Gauss–Newton method (GNM). Some recent applications of the LM algorithm are the speed control of an induction motor drive [36] and short-term wind speed prediction [37].

**Figure 2:**
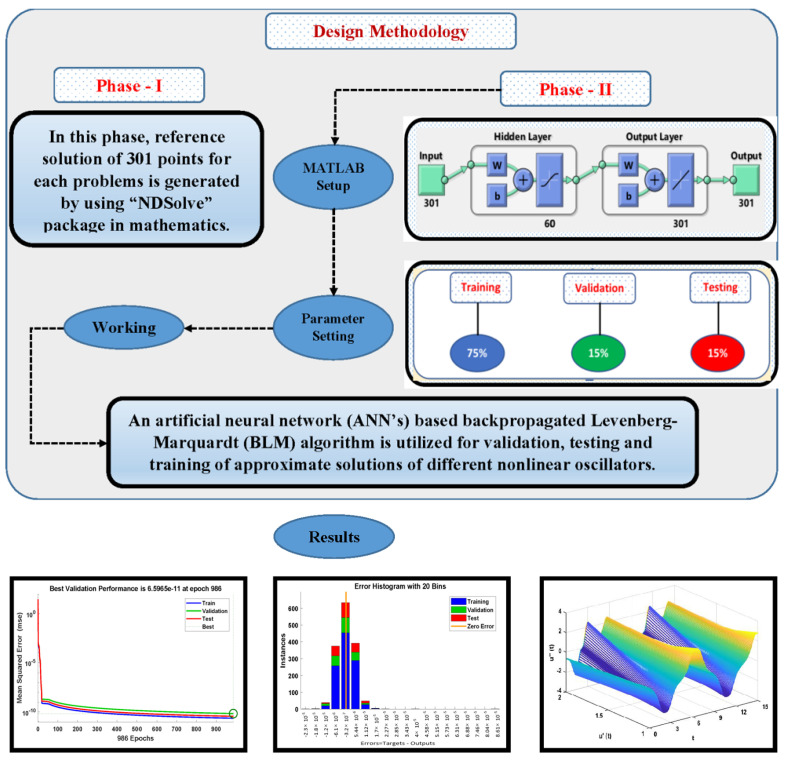
Working mechanism of NN-BLMA for solving strongly nonlinear oscillators.

Moreover, the implementation of NN-BLMA works in two phases. The detailed workflow of the design algorithm is presented in Figure 2.

- An initial data set is generated by using an analytical solution or calculating a numerical solution by using the Runge–Kutta method of order 4 (RK-4), with the ND Solve package in Mathematica.
- In the second phase, the BLM algorithm is executed by using “nftool” in the MATLAB package with appropriate settings of hidden neurons and testing data. Further, BLM uses a reference solution and implements the process of testing, training, and validation to obtain approximate solutions for different cases of nonlinear oscillators. Table 1 shows the parameter setting for the execution of the design scheme.

The NN-BLM algorithm has a simple structure and is easy to use in handling and processing the nonlinear problems. The NN-BLM algorithm is a gradient-free technique and its speed of convergence is much higher than that of other machine learning algorithms and state-of-the-art techniques. Further, to study the efficiency, stability, and convergence, the following performance indices are defined:

(3) $$\mathrm{MSE}=\frac{1}{m}\sum_{j=1}^{m}{\left(x_{j}\left(t\right)-{\hat{x}}_{j}\left(t\right)\right)}^{2},$$

(4) $$R^{2}=1-\frac{\sum_{j=1}^{m}{\left({\hat{x}}_{j}\left(t\right)-{\bar{x}}_{j}\left(t\right)\right)}^{2}}{\sum_{j=1}^{m}{\left(x_{j}\left(t\right)-{\bar{x}}_{j}\left(t\right)\right)}^{2}},$$

where $x_{j}$, ${\bar{x}}_{j}$, and ${\hat{x}}_{j}$ denote the reference, approximate, and mean of solution. *m* denotes the grid points. For the perfect modeling of approximate solutions, values of MSE and AE approach zero while the value of $R^{2}$ is 1.

## 3. Numerical Experimentation and Discussion

**Figure 3:**
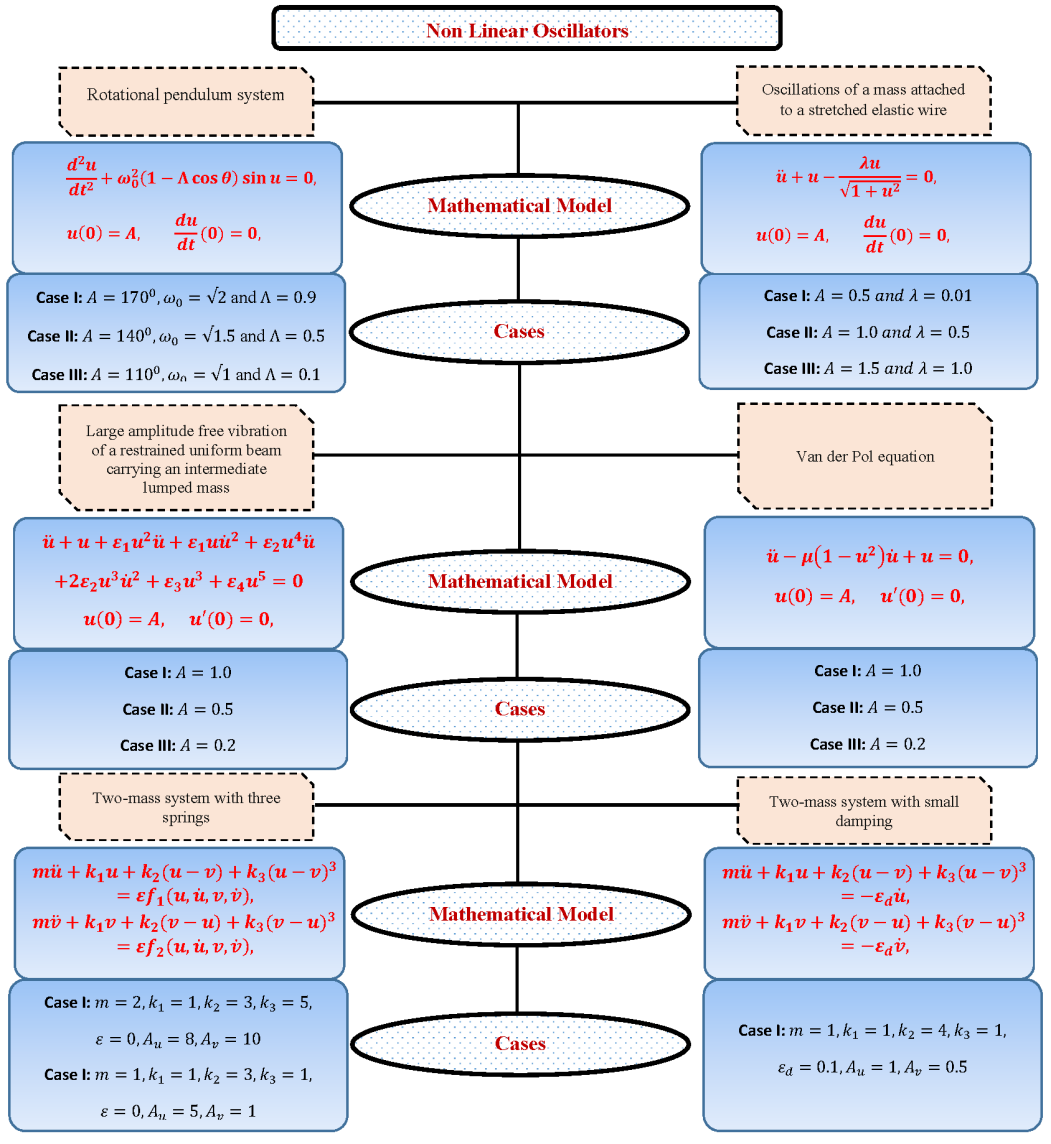
A general view of different cases of nonlinear oscillators discussed in this paper.

In order to study the performance and efficiency of the design algorithm, various cases of strongly nonlinear oscillators are formulated. Figure 3 presents a complete overview of the different problems and cases studied in this paper.

### 3.1. Rotational Pendulum

**Figure 4:**
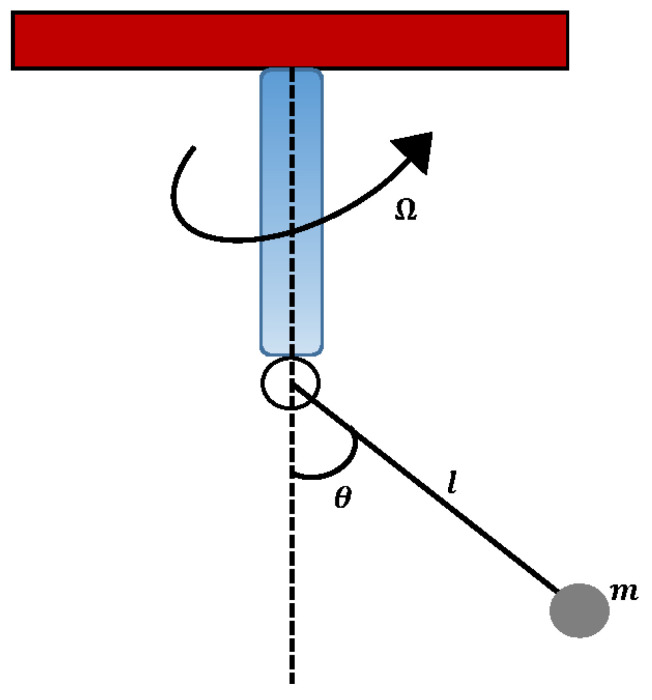
A schematic of a rotational simple pendulum.

Consider a mechanical model that represents a simple pendulum as shown in Figure 4. For large oscillation, at the neutral axis, a body of mass (m) is attached to a rotating base. This system’s equation for the motion is modeled as a second-order differential equation, which is given as [38]

(5) $$\frac{d^{2}u}{{dt}^{2}}+\omega_{0}^{2}\sin\left(u\right)\left(1-\Lambda \cos\left(u\right)\right)=0,\;u\left(0\right)=A\in \left(0^{\circ },180^{\circ }\right),\;\frac{du}{dt}\left(0\right)=0,$$

where *u* represents the angular displacement of the mass (m) in relation to time (t), and *A* denotes the initial amplitude of oscillation. $\omega_{0}^{2}$ and Λ are defined as

(6) $$\omega_{0}^{2}=\frac{g}{l},\;\Lambda =\frac{\Omega^{2}g}{l},$$

where ω, *l*, and *g* represent the angular velocity, length of weightless rod, and gravitational acceleration, respectively. A simple pendulum oscillates between the symmetric intervals [−a,a] when the revolver is pushed as a constant velocity. The range of values for Λ is assumed to be taken from (0,1). To study the effect of variations in Equation (5), the following cases are considered based on the increase in the amplitude of oscillation. Case I: $A=170^{\circ }$, $\omega_{0}=\sqrt{2}$, and Λ=0.9. Case II: $A=140^{\circ }$, $\omega_{0}=\sqrt{1.5}$, and Λ=0.5, Case III: $A=110^{\circ }$, $\omega_{0}=\sqrt{1}$, and Λ=0.1.

**Figure 5:**
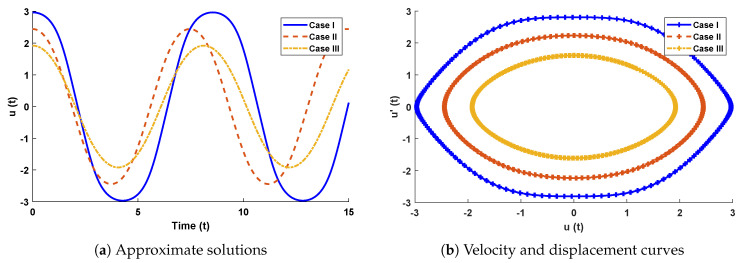
(**a**) Approximate solutions by the design scheme for different cases of rotational pendulum system, while (**b**) illustrates the phase plane between velocity and displacement of the system.

**Figure 6:**
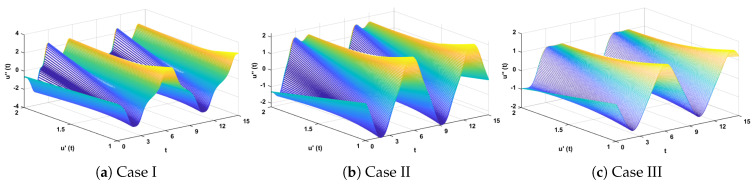
Three-dimensional plots to study the influence of time in velocity and acceleration of rotational pendulum system.

**Figure 7:**
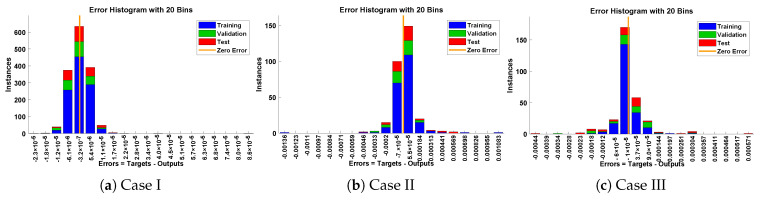
Error histogram analysis for each case of rotational pendulum.

**Figure 8:**
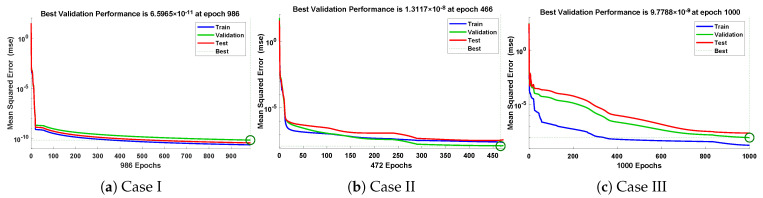
Convergence of performance function in terms of MSE for each case of problem 1.

**Figure 9:**
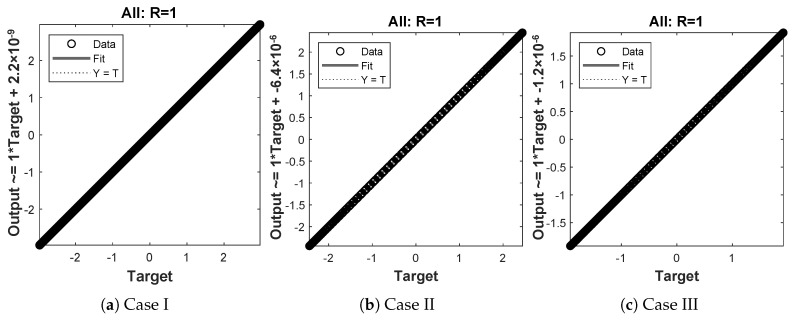
Regression analysis for different cases of problem 1.

In this problem, the influence of variations in different parameters of the rotational pendulum system has been investigated by using the NN-BLM algorithm. Approximate solutions obtained by the proposed algorithm are compared with He’s Energy Balance Method (HEBM) [39], as shown in Table 2. The results for displacement, velocity, and acceleration are given in Table 3 and graphically shown in Figure 5. Periodic results show that displacement decreases with the increase in time. To study the relation of velocity and acceleration with time, three-dimensional surface graphs were plotted and are shown in Figure 6. The results show that a decrease in the amplitude of oscillation causes a decrease in the velocity and acceleration of the pendulum. Further, to study the effectiveness of the solutions and the efficiency of the design algorithm, error histogram graphs and performance graphs were plotted, as shown in Figure 7 and Figure 8, respectively. The results obtained by the design algorithm overlap with the analytical solution with minimum absolute errors. The absolute error for each case lies around $10^{-5}$ to $10^{-7}$, $10^{-3}$ to $10^{-5}$, and $10^{-4}$ to $10^{-5}$, respectively. In addition, the values of performance function show the perfect modeling of solutions as they lie around $6.5965\times 10^{-11}$, $1.3117\times 10^{-8}$, and $9.7788\times 10^{-9}$, respectively. Statistics of validation, testing, and training of the reference solution are provided in Table 4. Regression analysis is given in Figure 9, which shows the accuracy of the proposed algorithm in calculating approximate solutions.

### 3.2. Oscillations of a Mass Attached to a Stretched Elastic Wire

**Figure 10:**
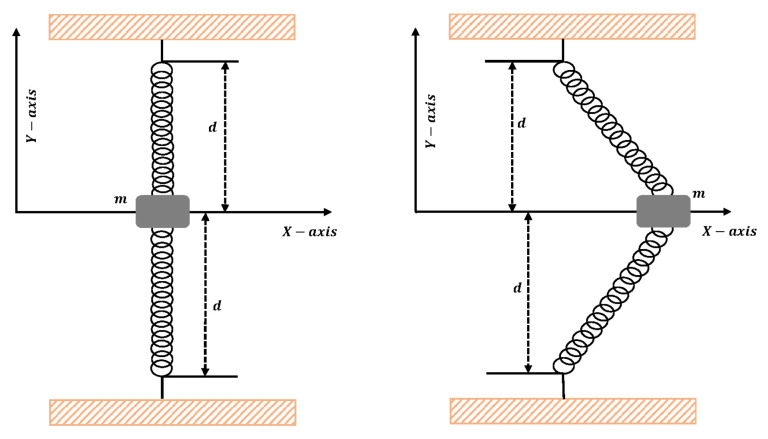
Mass attached to the center of elastic wire.

Consider the particle of mass (m) attached to the center of a stretched elastic wire, as shown in Figure 10. The one-dimensional equation of motion for the mass moving in the horizontal direction is given as [15]

(7) $$m\frac{d^{2}x}{d\tau^{2}}+2kx-\frac{2kax}{\sqrt{d^{2}+x^{2}}}=0,$$

subject to the initial conditions

(8) $$x\left(0\right)=B,\;\frac{dx}{dt}\left(0\right)=0,$$

and dimensionless variables are defined in terms of *u* and *t* as

(9) $$u=\frac{x}{d},\;t=\sqrt{\frac{2k}{m}}\tau ,$$

where *k* is the coefficient of stiffness and 2d denotes the length of the elastic wire. Substituting Equation (9) into Equation (7), we obtain

(10) $$\ddot{u}+u-\frac{\lambda u}{\sqrt{1+u^{2}}}=0\;\lambda \in \left(0,1\right],\;u\left(0\right)=A,\frac{du}{dt}\left(0\right)=0$$

where $\lambda =\frac{a}{b}$ and $A=\frac{B}{D}$. Further, the variations in *A* and λ are studied by considering the following cases. Case I: A=0.5 and λ=0.01, Case II: *A* = 1.0 and λ=0.5 and Case III: A=1.5 and λ=1.0.

**Figure 11:**
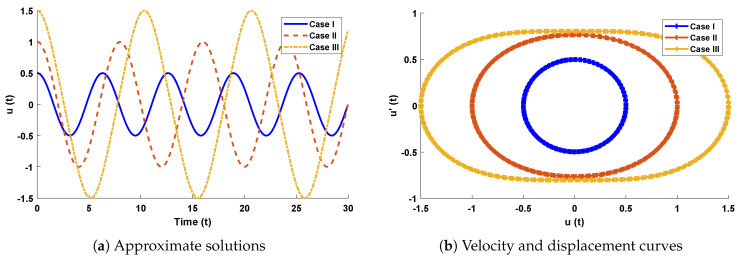
(**a**) Approximate solutions obtained by proposed algorithm for the system. (**b**) shows the phase plane between velocity and displacement of the stretched elastic wire.

**Figure 12:**
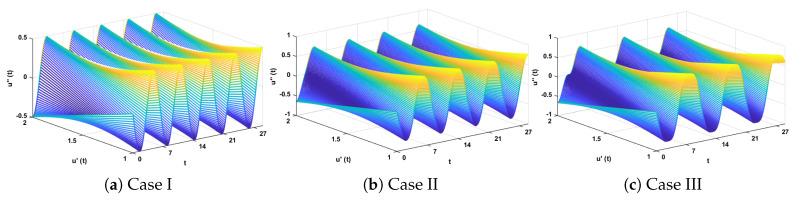
Three-dimensional plots to study the influence of time in velocity and acceleration of mass attached to a stretched elastic wire.

**Figure 13:**
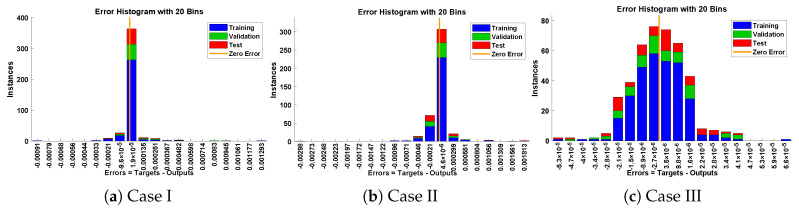
Error histogram analysis for each case of mass attached to stretched elastic wire.

**Figure 14:**
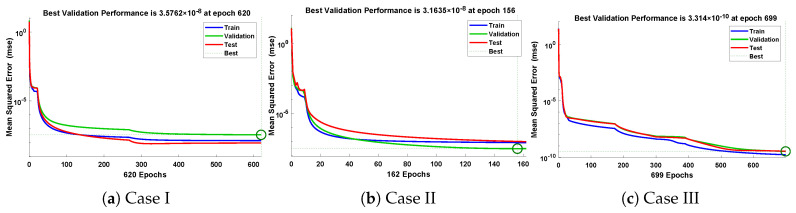
Convergence of performance function in terms of mean square error for each case of problem 2.

The design algorithm NN-BLMA is executed to study the influence of variations in oscillations of a mass attached to an elastic wire. Statistics of approximate solutions for angular displacement, velocity, and acceleration obtained by the proposed algorithm are given in Table 3. Approximate solutions and the phase plane curves between the angular velocity and displacement are shown in Figure 11. Moreover, from Figure 12, it can be observed that the amplitude of oscillations increases but the periodic curves decrease with an increase in time, which causes the decreases in angular displacement and velocity of the system. Further, to study the effectiveness of the solutions, convergence, error histogram, and regression analysis were carried out, as shown in Figure 13 and Figure 14, respectively. Numerical results show that the NN-BLM algorithm overlaps with the analytical solution with minimum absolute errors that lie around $10^{-3}$ to $10^{-5}$, $10^{-4}$ to $10^{-5}$, and $10^{-5}$ to $10^{-6}$, respectively. Table 4 represents the states of computational complexity of the design scheme in obtaining results for the nonlinear oscillator.

### 3.3. Large-Amplitude Free Vibration of a Restrained Uniform Beam Carrying an Intermediate Lumped Mass

**Figure 15:**
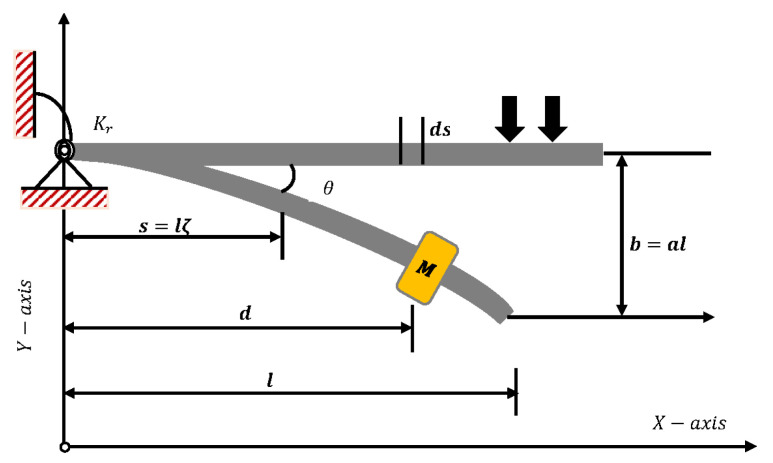
Geometry and coordinate system for a beam with rotational spring and a lumped mass.

In this problem, we consider a beam with uniform length *l* and mass (m) per unit length, hinged at the bottom of a rotational spring with stiffness $K_{r}$, as shown in Figure 15. The thickness of the beam is considered to be significantly smaller than the length of the beam; therefore, the effects of shear deformation and rotational inertia are neglected [20]. Inclination in the beam due to lumped mass in denoted by θ and displacement of the beam is given by a=b/l. Euler–Lagrange differential equations are used to derive a fifth-order Duffing-type model for the motion of the uniform beam carrying an intermediate lumped mass, which is given as

(11) $$\ddot{u}+u+\varepsilon_{1}u^{2}\ddot{u}+\varepsilon_{1}u{\dot{u}}^{2}+\varepsilon_{2}u^{4}\ddot{u}+2\varepsilon_{2}u^{3}{\dot{u}}^{2}+\varepsilon_{3}u^{3}+\varepsilon_{4}u^{5}=0,$$

with initial conditions

(12) $$u\left(0\right)=A,\;\frac{du}{dt}\left(0\right)=0,$$

where *u* denotes the dimensionless deflection of the beam at the tip, *A* is the maximum amplitude, and $\varepsilon_{1},\varepsilon_{2},\varepsilon_{3},\varepsilon_{4}$ are positive constants. Table 5 represents different cases of Equation (11) depending on the values of *A* and positive constants.

**Figure 16:**
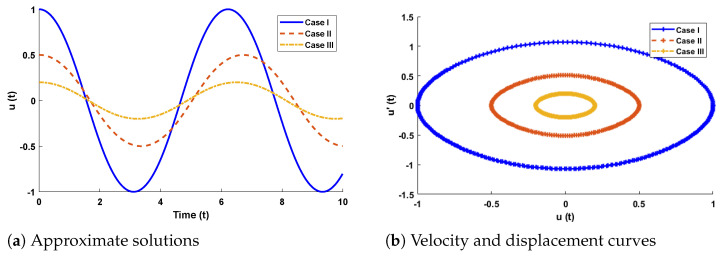
(**a**) Approximate solutions obtained by proposed algorithm for the system. (**b**) Phase plane analysis between velocity and displacement for mathematical model of restrained uniform beam carrying an intermediate lumped mass.

**Figure 17:**
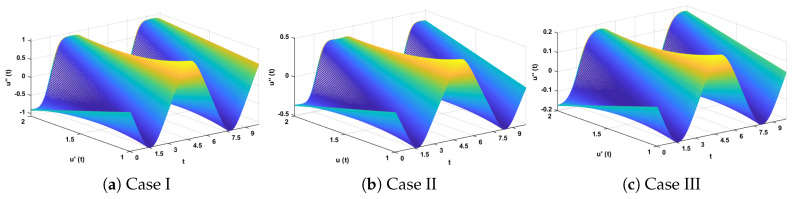
Three-dimensional plots to study the influence of time in velocity and acceleration of mathematical model given in Equation (6).

**Figure 18:**
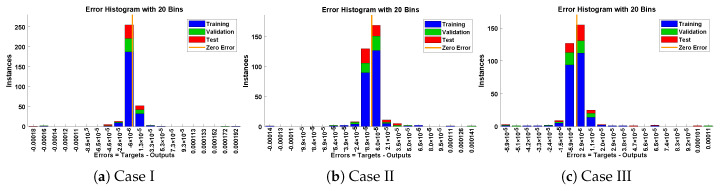
Error histogram analysis for each case of restrained uniform beam carrying an intermediate lumped mass.

**Figure 19:**
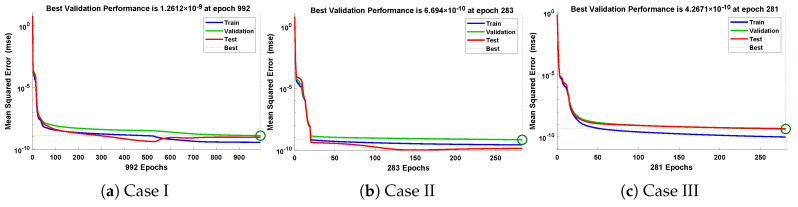
Convergence of performance function in terms of mean square error for each case of problem 3.

In this problem, the designed technique is applied to study the variations in amplitude and positive parameters on the deflection of uniform beam carrying an intermediate lumped mass. Table 3 lists the statistics of the approximate solutions in terms of displacement for each case of problem 3. Phase plane analysis of velocity against displacement is shown in Figure 16. The results show that deflection in the beam increases with an increase in the amplitude *A*. In addition, the oscillation in acceleration and velocity also increases, as shown in Figure 17. Absolute errors between targeted data and results obtained by the NN-BLM algorithm for different cases of Equation (11) are shown in Figure 18. The values of AE for each case lie around $10^{-4}$ to $10^{-6}$, $10^{-5}$ to $10^{-6}$, and $10^{-4}$ to $10^{-6}$, respectively. Table 6 presents the measure of convergence for each testing, validation, training, gradient, and mu. Complexity analysis in terms of the time taken by the system to achieve the desired results shows the robustness of the designed technique. It can be seen that the values for the gradient for each case lie around $9.97\times 10^{-8}$ to $9.98\times 10^{-8}$. Values of mu lie around $10^{-8}$ to $10^{-12}$. It can be seen from Figure 19 that the MSE for each case is approaching zero, which shows the accuracy of the proposed algorithm.

### 3.4. Van der Pol Equations

In this problem, we have considered van der Pol equations, which were introduced in 1920 by van der Pol to study the triode electric circuit and their self-sustained oscillations. The mathematical model for self-excited oscillations is given as

(13) $$\ddot{u}-\mu \left(1-u^{2}\right)\dot{u}+u=0,\;u\left(0\right)=A,\dot{u}\left(0\right)=B,$$

where μ is a scaling parameter that represents the length of damping and degree of nonlinearity. For μ, Equation (13) reduces to the equation of simple harmonic motion. Moreover, for u>1 and $\mu (1-u^{2})>0$, the system behaves as a damped one. To study the mathematical model of van der Pol oscillators, we have considered the following cases depending on different values of the scaling parameter, i.e., μ=0.1,1.0 and 10.

**Figure 20:**
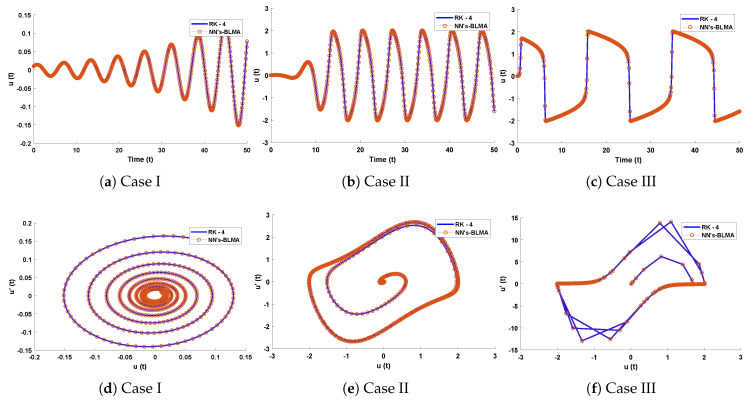
(**a**–**c**) Comparison of approximate solutions obtained by designed algorithm with RK-4. (**d**–**f**) show the analysis of phase plane between velocity and acceleration for van der Pol equation.

**Figure 21:**
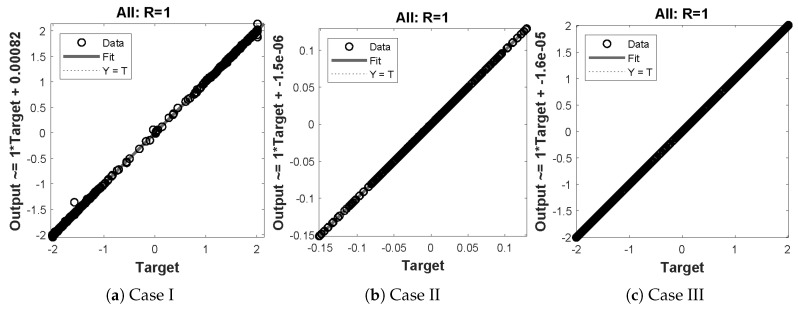
Regression analysis for different cases of problem 4.

In this problem, the effect of variations in μ has been investigated by the NN-BLM algorithm. Results obtained by the designed scheme for displacement, velocity, and acceleration are compared with RK-4, as shown in Figure 20. The results of the designed scheme overlap with the analytical solutions, with AEs that lie around $10^{-4}$ to $10^{-7}$. The performance of the fitness function in terms of mean square error is given in Table 7. The best values of the fitness function for each case are $2.357\times 10^{-9}$, $2.0835\times 10^{-7}$, and $9.5689\times 10^{-5}$ at epoch 100, 1000, and 340, respectively. Values of the gradient and mu for each case lie around $6.09\times 10^{3}$ to $9.95\times 10^{-8}$ and $10^{-3}$ to $10^{-8}$, respectively. Regression values for each case are exactly 1, as shown in Figure 21.

### 3.5. Two-Mass System with Three Springs

**Figure 22:**
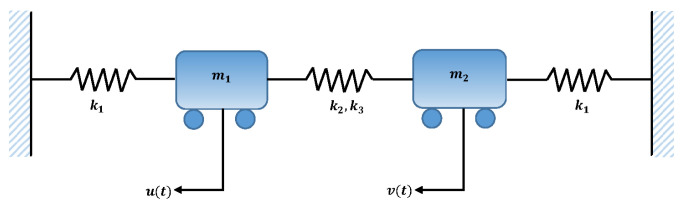
Schematic of two masses attached with three springs and fixed support.

In this problem, we consider a system of two equal masses (m) attached with fixed support and three springs with stiffness $k_{1}$, as shown in Figure 22. The equation for the motion of this system in generalized coordinates *u* and *v* is given as

(14) $$\left\{\begin{array}{c} m\ddot{u}+k_{1}u+k_{2}\left(u-v\right)+k_{3}{\left(u-v\right)}^{3}=\varepsilon f_{1}\left(u,\dot{u},v,\dot{v}\right),\\ m\ddot{v}+k_{1}v+k_{2}\left(v-u\right)+k_{3}{\left(v-u\right)}^{3}=\varepsilon f_{2}\left(u,\dot{u},v,\dot{v}\right), \end{array}\right.$$

subject to the initial conditions

(15) $$u\left(0\right)=A_{u},v\left(0\right)=A_{v},\;\frac{du}{dt}\left(0\right)=0,\;\frac{dv}{dt}\left(0\right)=0,$$

where $k_{2}$ and $k_{3}$ are the spring elasticity and cubic nonlinearity, respectively, while $\varepsilon f_{1}$ and $\varepsilon f_{2}$ are small nonlinearities. To briefly study the system, the following two cases are considered. Case I: $m=2.0,k_{1}=1.0,k_{2}=3.0,k_{3}=5.0,\varepsilon =0.0,A_{u}=8.0,A_{v}=10$. Case II: $m=1.0,k_{1}=1.0,k_{2}=3.0,k_{3}=1.0,\varepsilon =0.0,A_{u}=5.0,A_{v}=1.0$.

**Figure 23:**
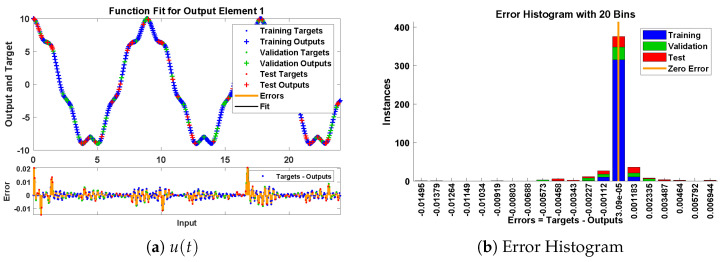

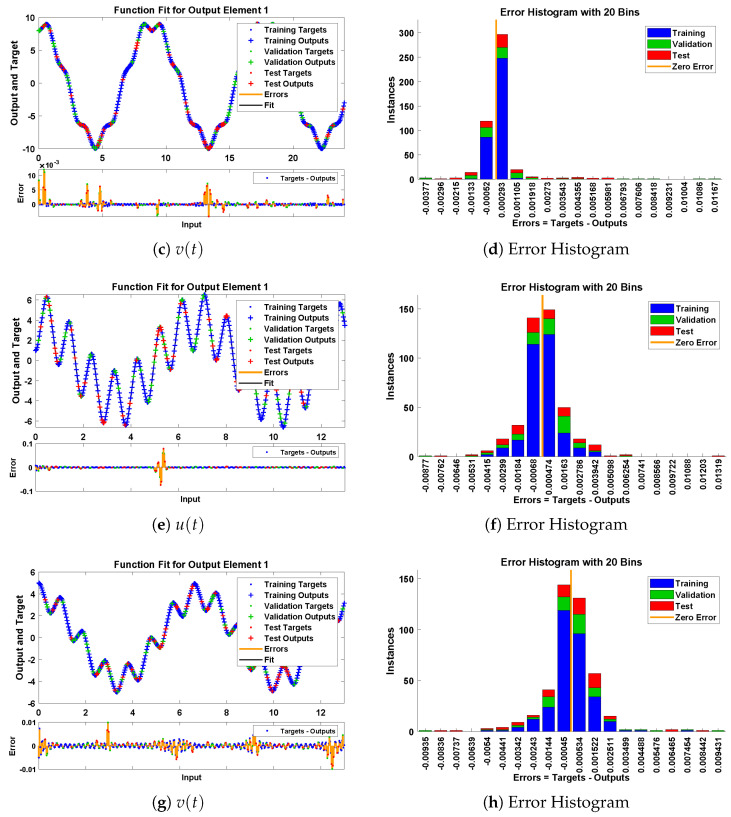
Approximate solution and histograms of u(t) and v(t) for Case I and II of problem 5.

**Figure 24:**
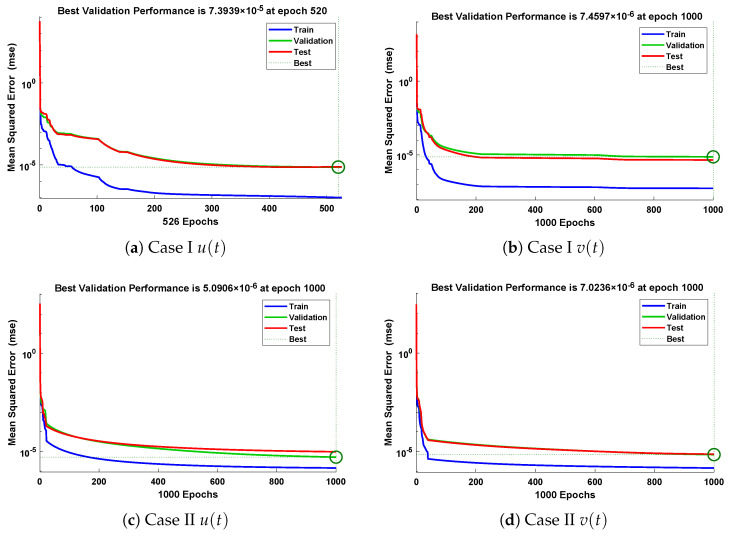
Analysis of performance function in terms of mean square error for different cases of problem 5.

**Figure 25:**
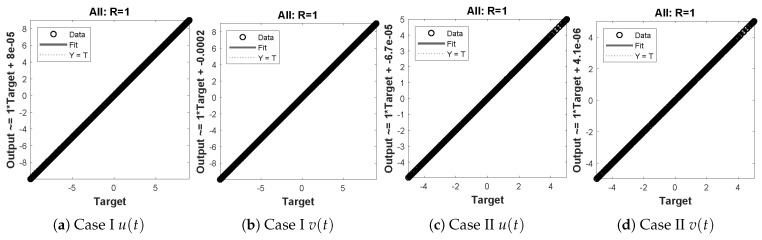
Regression plots for different cases of mass attached to three springs.

In this problem, the influence of variations in different parameters of mass attached to three springs has been investigated by using the NN-BLM algorithm. Approximate solutions obtained by the designed scheme are compared with targeted data generated from RK-4, as shown in Figure 23. It can be seen that the analytical solution overlaps with the approximate solutions, with minimum absolute errors that lie around $10^{-3}$ to $10^{-6}$. The results show that a decrease in mass increases the oscillations in the spring. Furthermore, the convergence plots for each case of problem 5 in terms of validation, testing, and training are shown in Figure 24. Statistics of the training parameters are provided in Table 8. Values of performance function for each case are $7.3939\times 10^{-6}$, $7.4597\times 10^{-6}$, $5.0906\times 10^{-6}$, and $7.0236\times 10^{-6}$ at epoch 520 and 1000, respectively. Analysis based on regression for each case is shown in Figure 25.

### 3.6. Two-Mass System with Small Damping

**Figure 26:**
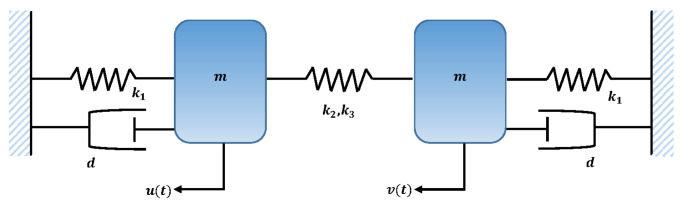
Schematic of two masses attached with three springs and fixed support.

In this problem, we consider the special case of problem 5 when there exists small damping. The model of the scenario is shown in Figure 26. The governing equations of motion for the system are given as

(16) $$\left\{\begin{array}{c} m\ddot{u}+k_{1}u+k_{2}\left(u-v\right)+k_{3}{\left(u-v\right)}^{3}=-\varepsilon_{d}\dot{u},\\ m\ddot{v}+k_{1}v+k_{2}\left(v-u\right)+k_{3}{\left(v-u\right)}^{3}=-\varepsilon_{d}\dot{v}, \end{array}\right.$$

with initial conditions

(17) $$u\left(0\right)=A_{u},\;v\left(0\right)=A_{v},\;\frac{du}{dt}\left(0\right)=0,\;\frac{dv}{dt}\left(0\right)=0$$

To find approximate solutions, we consider the case when $m=k_{1}=k_{3}=A_{u}=1,k_{2}=4,\varepsilon_{d}=0.1,A_{v}=0.5$.

**Figure 27:**
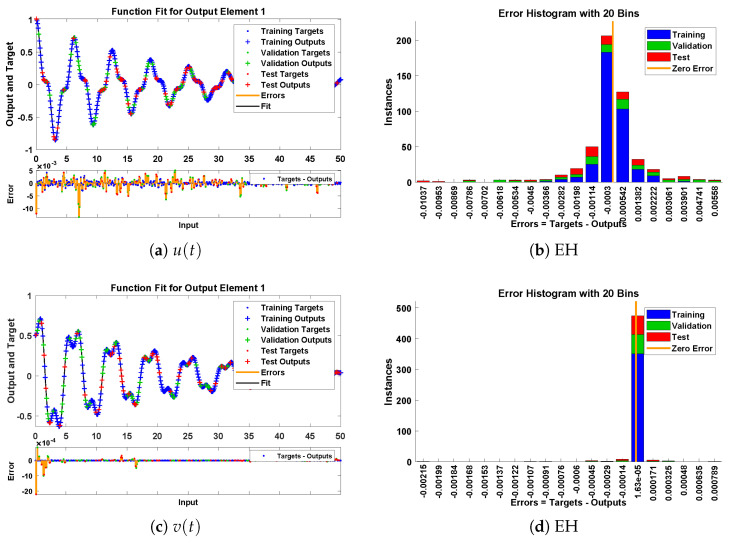
Approximate solution and histograms of u(t) and v(t) for problem 6.

**Figure 28:**
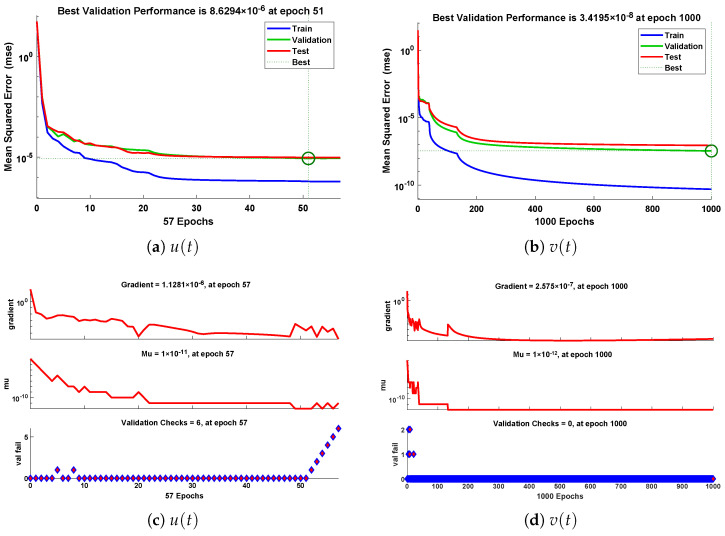
Performance state of training parameters and convergence of fitness function for example 6.

The designed scheme is exploited to determine the fitting of the approximate solutions with numerical results. The curve fitting of the approximate solutions by NN-BLMA for example 6 is plotted in Figure 27. The results are in good agreement with the analytical solutions as the absolute errors of u(t) and v(t) lie around $10^{3}$ to $10^{5}$, respectively. The performance of the objective function in terms of MSE for obtaining the best fitting is shown in Figure 28. The best validated performance of MSE is $8.6294\times 10^{-6}$, $3.4195\times 10^{-8}$, respectively. It is observed that the value of regression is one, which reflects the accuracy of the solutions obtained by the designed algorithm.

## 4. Conclusions

In this paper, an intelligent technique based on artificial neural networks is utilized to investigate the mathematical models of various nonlinear oscillators arising in physics, mechanics, and applied mathematics, such as a rotational pendulum system, mass attached to an elastic wire, a uniform beam carrying an intermediate lumped mass, van der Pol equation, a two-mass system with three springs, and a two-mass system with small damping. Furthermore, soft computing based on supervised learning of neural networks in used to calculate the displacements, velocity, and acceleration of nonlinear oscillators under the influence of different variations. A reference solution is generated by using the RK-4 method, which is then evaluated by the training, testing, and validation process of the Levenberg–Marquardt algorithm. The results obtained by the proposed algorithm are compared with He’s Energy Balance Method, Homotopy Analysis Method, Residue Harmonic Balance Method, and Homotopy Perturbation Method. Extensive graphical and statistical analysis shows that the designed algorithm is accurate and efficient as the approximate solutions overlap with the analytical solutions, with minimum absolute errors as compared to the state-of-the-art techniques. In addition, the values of performance indicators are approaching zero, which shows the perfect modeling of the results. 

## Figures and Tables

**Table 1:** Parameter setting for the implementation of the designed NN-BLM algorithm.

Testing	Training	Valiation	Hidden Neurons	Max. Ilteration	Max. Validation Fails	Performance Function
75%	15%	15%	60	1000	6	Mean Square Error

**Table 2:** Comparison of approximate solutions obtained by NN-BLM algorithm with He’s Energy Balance Method, Homotopy Analysis Method, Residue Harmonic Balance Method, and Homotopy Perturbation Method.

	Problem 1			Problem 2			Problem 3			Problem 5		
**t**	**Exact**	**HEBM**	**NN-BLMA**	**Exact**	**HAM**	**NN-BLMA**	**Exact**	**RHBM**	**NN-BLMA**	**Exact**	**HPM**	**NN-BLMA**
0	2.96706	3.01652	2.96706	1.5	1.5	1.5	1	1	1	8	7.999	8
3	−2.15119	−2.25409	−2.15119	−0.33557	−0.33647	−0.33557	−0.99448	−0.99442	−0.99448	−5.69251	−5.69215	−5.69251
6	−1.13609	−1.13986	−1.13609	−1.27728	−1.218	−1.27728	0.97792	0.97364	0.97792	−5.01246	−5.01245	−5.01246
9	2.89554	2.99654	2.89554	0.97753	0.96723	0.97753	−0.95033	−0.95047	−0.95033	8.10448	8.10448	8.10448
12	−2.69881	−2.831	−2.69881	0.72292	0.722911	0.72292	0.91174	0.91177	0.91174	−6.08405	−6.08401	−6.08405
15	0.12032	0.12087	0.12032	−1.4209	−1.42119	−1.4209	−0.86222	−0.86245	−0.86222	−4.07551	−4.07555	−4.07551

**Table 3:** Approximate solutions for angular displacements of problems 1, 2, and 3.

	Problem 1			Problem 2			Problem 3		
**t**	**Case I**	**Case II**	**Case III**	**Case I**	**Case II**	**Case III**	**Case I**	**Case II**	**Case III**
0	2.96706	2.44346	1.91986	0.5	1	1.5	1	0.5	0.2
3	−2.15119	−2.07124	−1.36661	−0.49397	−0.70011	−0.33557	−0.99448	−0.47592	−0.19447
6	−1.13609	0.8869	−0.14399	0.47604	0.00452	−1.27728	0.97792	0.40334	0.17801
9	2.89554	0.71232	1.53846	−0.44663	0.69344	0.97753	−0.95033	−0.2829	−0.15114
12	−2.69881	−1.97792	−1.90429	0.40645	−0.99995	0.72292	0.91174	0.12156	0.11487
15	0.12032	2.4389	1.16415	−0.35648	0.70671	−1.4209	−0.86222	0.06023	−0.07096

**Table 4:** Statistics of performance measures for obtaining solutions to problems 1 and 2.

		Mean Square Error							
**Case**	**Neurons**	**Training**	**Validation**	**Testing**	**Gradient**	**Mu**	**Epochs**	**Regression**	**Time**
I	60	$2.22\times 10^{-11}$	$6.60\times 10^{-11}$	$3.49\times 10^{-11}$	$9.98\times 10^{-8}$	$1.00\times 10^{-11}$	985	1	2 s
II	60	$2.67\times 10^{-8}$	$1.31\times 10^{-8}$	$3.72\times 10^{-8}$	$6.64\times 10^{-6}$	$1.00\times 10^{-11}$	472	1	0.01 s
III	60	$1.93\times 10^{-9}$	$9.78\times 10^{-9}$	$2.55\times 10^{-8}$	$2.08\times 10^{-6}$	$1.93\times 10^{-9}$	1000	1	2 s
I	60	$1.36\times 10^{-8}$	$3.58\times 10^{-8}$	$9.19\times 10^{-9}$	$9.98\times 10^{-8}$	$1.00\times 10^{-10}$	620	1	0.03 s
II	60	$8.57\times 10^{-8}$	$3.16\times 10^{-8}$	$1.04\times 10^{-7}$	$3.23\times 10^{-6}$	$1.00\times 10^{-9}$	162	1	1s
III	60	$1.68\times 10^{-10}$	$3.31\times 10^{-10}$	$3.40\times 10^{-10}$	$9.92\times 10^{-8}$	$1.00\times 10^{-9}$	699	1	0.02 s

**Table 5:** Values of parameters involved in mathematical model of restrained uniform beam carrying an intermediate lumped mass.

Cases	Amplitude (A)	$\varepsilon_{1}$	$\varepsilon_{2}$	$\varepsilon_{3}$	$\varepsilon_{4}$
I	1	0.326845	0.129579	0.232598	0.087584
II	0.5	1.642033	0.913055	0.313561	0.204297
III	0.2	4.051486	1.665232	0.281418	0.149677

**Table 6:** Statistics of performance measures for obtaining solutions to problems 3 and 4.

		Mean Square Error							
**Case**	**Neurons**	**Training**	**Validation**	**Testing**	**Gradient**	**Mu**	**Epochs**	**Regression**	**Time**
I	60	3.67 × 10${}^{-10}$	1.26 × 10${}^{-9}$	9.37 × 10${}^{-10}$	9.98 × 10${}^{-8}$	1.00 × 10${}^{-11}$	992	1	0.02 s
II	60	2.61 × 10${}^{-10}$	6.69 × 10${}^{-10}$	1.35 × 10${}^{-10}$	9.97 × 10${}^{-8}$	1.00 × 10${}^{-11}$	283	1	0.01 s
III	60	9.69 × 10${}^{-11}$	4.27 × 10${}^{-10}$	4.63 × 10${}^{-10}$	9.97 × 10${}^{-8}$	1.00 × 10${}^{-11}$	281	1	0.01 s
I	60	3.76 × 10${}^{-10}$	2.36 × 10${}^{-9}$	3.16 × 10${}^{-9}$	9.95 × 10${}^{-8}$	1.00 × 10${}^{-12}$	100	1	0.005 s
II	60	5.27 × 10${}^{-8}$	2.08 × 10${}^{-7}$	2.99 × 10${}^{-7}$	1.72 × 10${}^{-5}$	1.00 × 10${}^{-9}$	1000	1	2 s
III	60	1.02 × 10${}^{-4}$	9.57 × 10${}^{-4}$	6.91 × 10${}^{-4}$	6.09 × 10${}^{-3}$	1.00 × 10${}^{-8}$	340	1	0.02 s

**Table 7:** Approximate solutions for displacement of problems 4, 5, and 6.

	Problem 4			Problem 5			Problem 6		
**t**	**Case I**	**Case II**	**Case III**	**Case I**		**Case II**		**Case I**	
				u(t)	v(t)	u(t)	v(t)	u(t)	v(t)
0	0.01	0.01	0.01	8	10	5	1	1	0.5
3	−0.0099	−0.02498	1.50214	−5.69251	−3.7239	−3.07566	−4.7436	−0.84116	−0.42692
6	0.00925	−0.00824	0.44609	−5.01246	−3.13545	2.24812	4.25251	0.67892	0.36974
9	−0.00791	0.42076	−1.83206	8.10448	9.83682	−2.90897	1.08233	−0.51973	−0.327
12	0.00571	−1.03501	−1.56265	−6.08405	−4.53945	0.00641	1.33001	0.37165	0.29367
15	−0.00244	1.39666	−1.03589	−4.07551	−2.75077	1.67698	−0.64469	−0.24233	−0.26364

**Table 8:** Statistics of performance measures by NN-BLMA for obtaining solutions to problems 5 and 6.

		Mean Square Error							
**Case**	**Neurons**	**Training**	**Validation**	**Testing**	**Gradient**	**Mu**	**Epochs**	**Regression**	**Time**
I	60	1.03 × 10${}^{-7}$	7.39 × 10${}^{-6}$	7.68 × 10${}^{-6}$	2.63 × 10${}^{-5}$	1.00 × 10${}^{-10}$	526	1	0.06 s
	60	5.48 × 10${}^{-8}$	7.46 × 10${}^{-6}$	4.58 × 10${}^{-6}$	2.17 × 10${}^{-5}$	1.00 × 10${}^{-9}$	1000	1	2 s
II	60	1.43 × 10${}^{-6}$	5.09 × 10${}^{-6}$	9.57 × 10${}^{-6}$	1.54 × 10${}^{-4}$	1.00 × 10${}^{-8}$	1000	1	2 s
	60	1.46 × 10${}^{-6}$	7.02 × 10${}^{-6}$	7.38 × 10${}^{-6}$	2.55 × 10${}^{-5}$	1.00 × 10${}^{-8}$	1000	1	2 s
I	60	4.96 × 10${}^{-11}$	3.42 × 10${}^{-8}$	8.76 × 10${}^{-8}$	2.58 × 10${}^{-7}$	1.00 × 10${}^{-12}$	1000	1	2 s
	60	6.18 × 10${}^{-7}$	8.63 × 10${}^{-6}$	9.52 × 10${}^{-6}$	1.13 × 10${}^{-6}$	1.00 × 10${}^{-11}$	57	1	0.001 s

## Data Availability

The data that support the findings of this study are available from the corresponding author upon reasonable request.
